# A Higher Abundance of *Actinomyces* spp. in the Gut Is Associated with Spontaneous Preterm Birth

**DOI:** 10.3390/microorganisms11051171

**Published:** 2023-04-29

**Authors:** Hong-Ren Yu, Ching-Chang Tsai, Julie Y. H. Chan, Wei-Chia Lee, Kay L. H. Wu, You-Lin Tain, Te-Yao Hsu, Hsin-Hsin Cheng, Hsin-Chun Huang, Cheng-Hsieh Huang, Wen-Harn Pan, Yao-Tsung Yeh

**Affiliations:** 1 Department of Pediatrics, Chang Gung Memorial Hospital-Kaohsiung Medical Center, Graduate Institute of Clinical Medical Science, Chang Gung University College of Medicine, Kaohsiung 83301, Taiwan; 2 Department of Obstetrics and Gynecology, Kaohsiung Chang Gung Memorial Hospital, Kaohsiung 83301, Taiwan; 3 Institute for Translational Research in Biomedicine, Kaohsiung Chang Gung Memorial Hospital, Kaohsiung 83301, Taiwan; 4 Division of Urology, Kaohsiung Chang Gung Memorial Hospital, and Chang Gung University College of Medicine, Kaohsiung 83301, Taiwan; 5 Ph.D. Program in Environmental and Occupational Medicine, College of Medicine, Kaohsiung Medical University and National Health Research Institutes, Kaohsiung 83130, Taiwan; 6 Aging and Disease Prevention Research Center, Fooyin University, Kaohsiung 83130, Taiwan; 7 BioMed Analysis Center, Fooyin University Hospital, Pingtung 92847, Taiwan; 8 Department of Medical Laboratory Sciences and Biotechnology, Fooyin University, Kaohsiung 83130, Taiwan; 9 Institute of Biomedical Sciences, Academia Sinica, Taipei 11529, Taiwan

**Keywords:** spontaneous preterm birth, maternal microbiome, *Actinomyces* spp., glycan biosynthesis

## Abstract

Preterm birth is a major challenge in pregnancy worldwide. Prematurity is the leading cause of death in infants and may result in severe complications. Nearly half of preterm births are spontaneous, but do not have recognizable causes. This study investigated whether the maternal gut microbiome and associated functional pathways might play a key role in spontaneous preterm birth (sPTB). Two hundred eleven women carrying singleton pregnancies were enrolled in this mother-child cohort study. Fecal samples were freshly collected at 24–28 weeks of gestation before delivery, and the 16S ribosomal RNA gene was sequenced. Microbial diversity and composition, core microbiome, and associated functional pathways were then statistically analyzed. Demographic characteristics were collected using records from the Medical Birth Registry and questionnaires. The result showed that the gut microbiome of mothers with over-weight (BMI ≥ 24) before pregnancy have lower alpha diversity than those with normal BMI before pregnancy. A higher abundance of *Actinomyces* spp. was filtered out from the Linear discriminant analysis (LDA) effect size (LEfSe), Spearman correlation, and random forest model, and was inversely correlated with gestational age in sPTB. The multivariate regression model showed that the odds ratio of premature delivery was 3.274 [95% confidence interval (CI): 1.349; *p* = 0.010] in the group with over-weight before pregnancy with a cutoff Hit% > 0.022 for *Actinomyces* spp. The enrichment of *Actinomyces* spp. was negatively correlated with glycan biosynthesis and metabolism in sPTB by prediction from the Investigation of Communities by Reconstruction of Unobserved States (PICRUSt) platform. Maternal gut microbiota showing a lower alpha diversity, increased abundance of *Actinomyces* spp., and dysregulated glycan metabolism may be associated with sPTB risk.

## 1. Introduction

Preterm delivery is defined as the live delivery of babies before the 37th week of pregnancy. According to estimates, the incidence of preterm births is about one-tenth of all newborns, with 15 million preterm babies being born each year worldwide [1]. Prematurity is the most common cause of neonatal morbidity and mortality worldwide [2]. Surviving children are at risk of adverse consequences, including hypoxic-ischemic encephalopathy, patent ductus arteriosus, necrotizing enterocolitis, bronchopulmonary dysplasia, retinopathy, cognitive impairments, and learning disabilities [2,3]. The underlying etiology of preterm birth (PTB) is multifactorial and remains incompletely understood. The risk factors for preterm delivery can be classified as maternal and pregnancy-related factors [3]. Maternal factors associated with preterm birth include ethnicity, lower socioeconomic status, age, smoking, and BMI. The major pregnancy-related risk factors for preterm birth include the use of assisted reproductive technology, multiple gestation pregnancies, and intrauterine infection. However, nearly half of the preterm births are spontaneous, without known associated surgical or medical causes [4]. Hence, the mechanism of spontaneous preterm birth (sPTB) needs to be clarified.

Intrauterine infection is a critical mechanism that contributes to premature birth [5]. The mechanisms by which intrauterine infections lead to preterm labor are related to the activation of the innate immune system [6]. Microorganisms are recognized by pattern-recognition receptors, which trigger the release of proinflammatory cytokines such as interleukin (IL)-8, IL-1β, and tumor necrosis factor (TNF)-α. Microbial endotoxins and proinflammatory cytokines then evoke the production of prostaglandins and extracellular matrix-degrading enzymes. Prostaglandins can enhance uterine contractility, while the degradation of the extracellular matrix of the fetal membranes leads to premature rupture of membranes [6]. It is known that chorioamnionitis is more common in preterm labor than in full-term labor [7]. Multiple studies have reported an association between preterm labor/delivery, various genital tract infections, and the colonization of certain bacteria, including *group B streptococci*, *Chlamydia trachomatis*, *Neisseria gonorrhea*, *syphilis*, *Trichomonas vaginalis*, and *Ureaplasma* spp. [8,9,10,11]. A positive culture for these species correlates with the presence of histologic chorioamnionitis; however, causal relationships between most of these infections and PTB have not been proven and remain controversial [10,12]. One explanation for these inconsistent findings may be that intrauterine infection is sometimes difficult to detect using conventional culture techniques [13]. Advances in molecular analysis methods have provided new insights into the role of microbe-host interactions during pregnancy and PTB risk [14]. Therefore, the relationship between microorganisms and preterm birth is changed from the original pathogenic microorganisms causing PTB to the microbiome and relevant metabolites affecting maternal and fetal health.

The microbiome modulates the physiological and metabolic homeostasis of the host. From histological and molecular biological studies, chorioamnionitis and fetal membrane inflammation have been observed to be closely related to preterm birth [15,16,17]. The vaginal microbiome is the most widely studied microbiome associated with PTB. The vaginal microbiota is dominated by a variety of *Lactobacillus* spp. These *Lactobacillus* spp. can produce lactic acid, acidify the vaginal environment, and affect the growth and colonization of other pathogens [18]. Studies have shown that the normal vaginal microbiota varies by ethnicity. Asians tend to carry *Lactobacillus iners*, Caucasians tend to carry *L. crispatus*, while Hispanics and African-Americans tend to carry a variety of bacteria but only a few *Lactobacillus* species [19,20,21]. The vaginal microbiome is relatively stable during pregnancy [15]. One study demonstrated that the carriage of vaginal *L. crispatus* is a protective factor for PTB in pregnant women of European ancestry [22]. Another study pointed out that in both Caucasian and African-American gravidae, the decreased abundance of *L. crispatus* and increased abundance of *Prevotella* are related to PTB [23]. In another study in the UK, the dominance of *L. iners* at 16 weeks of pregnancy was associated with PTB [24]. Both the risk of PTB and the composition of the vaginal microbiome in the normal population are ethnicity-dependent; however, these inconsistent results need to be validated in future studies.

Reports on the shift in gut microbiomes by gestational age are also inconsistent. For example, several studies have shown that with an increase in gestational age, the diversity and composition of the gut microbiome of pregnant women are still relatively stable [25,26]. In contrast, another group found that, compared with the first trimester, the phylogenetic diversity of the gut microbiome decreased during the third trimester of pregnancy [27]. They also observed that the relative abundances of *Proteobacteria* and *Actinobacteria* increased in 69% and 57% of women in the third trimester, respectively [27]. The association between the gut microbiome of pregnant women and preterm delivery has been less studied. Dahl et al. found that, compared with 102 Norwegian women who gave birth at term, the intestinal flora of 19 preterm women showed a lower abundance of *Bifidobacterium*, *Streptococcus*, and families in the *Clostridium* order on the fourth day after delivery [28]. Shiozaki et al. showed that, compared with 10 women who gave birth at term, the abundance of several species of *Clostridium* and *Bacteroides* in 10 women who delivered preterm was reduced before delivery in Japan [29]. Although these studies provide links between gut microbes, inflammation, and mediating the risk of sPTB, more investigations are needed to obtain conclusive results and reflect these into routine clinical practice. Furthermore, significant geographic and ethnic differences remain to be clarified.

Identifying women who are at risk of preterm delivery allows the provision of appropriate preventive interventions that can improve neonatal outcomes. However, predicting preterm delivery is difficult because of its diverse etiology. To date, no specific microbiome component has been confirmed to be reliably associated with preterm birth across ethnicities. Our hypothesis was that the maternal gut microbiome is closely related to sPTB. This study aims to investigate whether the maternal gut microbiome and its associated functional pathways play a key role in sPTB. Since the geographic environment, eating habits, and ethnic differences can affect the gut microbiome, it is necessary to investigate the relationship between the gut microbiome and preterm birth in localized settings.

## 2. Materials and Methods

### 2.1. Study Population

This pregnancy–birth cohort study was conducted at an antenatal clinic in Kaohsiung Chang Gung Memorial Hospital, a medical center in southern Taiwan. The participants were enrolled between August 2019 and July 2020. All participants provided written informed consent prior to enrolment. Pregnant Taiwanese women without clinical complications (diabetes, eclampsia, or preeclampsia) who intend to deliver their babies at our hospital were invited. Maternal blood and stool samples were collected at 24–28 weeks of gestation during the regular antenatal visits of these women. The fecal samples were collected by a noncommercial collection tube with DNA Stabilizer [30] at home and fecal DNA would be purified by QIAmp stool mini kit (Qiagen, Germany). For serum and plasma collection, the blood sample was collected in a tube with and without heparin. After centrifugation, samples were immediately transferred to the laboratory and stored at −80 °C before analysis. The study protocol was approved by the Institutional Review Board of the Chang Gung Memorial Hospital, Kaohsiung Medical Center (IRB: 201900161A3C603).

### 2.2. Questionnaire

A face-to-face interview was conducted with the participants in their second trimester to collect maternal demographic and socioeconomic characteristics (such as age, ethnicity, and education), disease history, and lifestyle during pregnancy (including alcohol consumption and smoking, and home and external environments such as humidity, among others). A unified questionnaire was used to obtain demographic information. The mother’s pre-pregnancy anthropometric measurements were self-reported.

### 2.3. Food Intake Frequency Evaluation

Food consumption frequency (times/week) was evaluated using a food frequency questionnaire [31]. Twelve high-quality food categories (vegetables, fruits, milk, yogurt, meat, poultry, pork, beef, fish, soy milk, soy products, and eggs) and nine low-quality food categories (fried food, ice cream, sugary and high-fat foods, high-fat snacks, instant noodles, sugar drinks, shaved ice desserts, candy, and chocolate) were included in the questionnaire.

### 2.4. Microbial Analysis

#### 2.4.1. Microbial DNA Extraction and Sequencing

Microbial DNA was extracted from stool samples using a QIAmp DNA Stool Mini Kit (Qiagen, Germany), according to the manufacturer’s protocol. The V3–V4 region of the bacterial 16S rDNA gene was amplified using PCR (thermocycling conditions: 95 °C for 3 min, followed by 30 cycles at 95 °C for 30 s, 55 °C for 30 s, 72 °C for 45 s, and a final extension at 72 °C for 10 min). The following primers were used: 5′-TCG TCG GCA GCG TCA GAT GTG TAT AAG AGA CAG CCT ACG GGN GGC WGC AG-3′ (forward) and 5′-GTC TCG TGG GCT CGG AGA TGT GTA TAA GAG ACA GGA CTA CHV GGG TAT CTA ATC C-3′ (reverse) [32]. The predicted amplicon size was approximately 550 bp, which was verified using a Fragment Analyzer system (Agilent Technologies Inc.). The amplified DNA was then purified using KAPA Pure Beads (Roche) according to the manufacturer’s protocol. Library index PCR was performed using an Illumina Nextera XT DNA Library Preparation Kit Set A. Amplicons were purified using KAPA Pure Beads DNA (Roche) according to the manufacturer’s instructions and quantified using a Qubit 4 fluorometer (Thermo Fisher Scientific, Wilmington, DE, USA). Purified amplicons were then pooled in 10 μM and paired-end sequences (2 × 300) were obtained using an Illumina MiSeq platform (MiSeq v3 600 cycle kit) according to the standard protocol.

#### 2.4.2. Bioinformatic Analysis

More than 100,000 raw sequencing reads obtained from the Illumina MiSeq analyzer were picked from each sample and used to compare the community composition and structure among samples after reads’ filtering by trimming left and right primer (10 bps) and Removing Phix DNA by the CLC genomics workbench (v10.1.1, Qiagene, Germany). The operational taxonomy units (OTUs) tables with 97%-similarity were generated according to Greengene (v13.8) database by the CLC genomics workbench. The alpha (i.e., Shannon Diversity index) and beta (Weighted UniFrac distance) diversity using the above-generated OTUs were further analyzed by the MicrobiomeAnalyst (https://www.microbiomeanalyst.ca) accessed on 1 January 2022. The linear discriminant analysis (LDA) effect size (LEfSe) analysis for critical bacteria in preterm was performed by the Galaxy Website (http://huttenhower.sph.harvard.edu/galaxy/) accessed on 1 January 2022. The threshold on the logarithmic LDA score for discriminative features was LDA > 2.0. Spearman’s correlation, which was performed by the R language (v4.2.3), was used to analyze correlated bacteria with delivery weeks. As for PICRUSt analysis, all predicted gene families and pathways were compared to those from metagenome sequencing in terms of their KEGG annotations via Galaxy Website. The resultant OTU table was normalized with inferred 16S rRNA gene copy numbers and predicted microbial metagenomes using a script provided by PICRUSt (ver. 1.0.0) on the Galaxy website according to the Kyoto Encyclopedia of Genes and Genomes (KEGG) functional pathways database and further analyzed by Statistical Analysis of Metagenomic Profiles (STAMP) software version 2.1.3. Furthermore, the Random Forest analyses using microbiota composition at baseline as the independent variable and preterm and BMI as dependent variables to identify taxa that might be differently abundant in the gut of preterm birth and maternal body weight. This method was conducted by the MicrobiomeAnalyst.

### 2.5. Statistics

The raw sequencing reads were assigned to the classification hierarchy using the CLC genomics workbench (v10.1.1) for the principal component analysis (PCoA). The raw read count was normalized to the total reads, and PCoA was performed using the CLC genomics workbench. The mean values of alpha diversity were compared between each group by the unpaired student *t*-test. Data are expressed as the mean ± standard error of the mean. The beta diversity of microbial composition was analyzed by the PERMANOVA test using the CLC genomics workbench. The unpaired t test was used when two groups were analyzed. A one-way ANOVA with the Bonferroni test was used when more than two groups were analyzed. The correlation between gestational age and maternal gut microbiome was analyzed with Spearman’s correlation test. Multivariate regression models were characterized through the area under the receiver operating characteristic (ROC) curve (AUC). The Benjamini–Hochberg false discovery rate (FDR) correction for multiple comparisons was applied separately. FDR (q-value) < 0.05 was considered significant. The LEfSe analysis was examined by the Wilcoxon test. Comparison of means, multivariant regression model, and ROC curve were performed using SPSS (version 25.0; SPSS Inc., Chicago, IL, USA).

## 3. Results

### 3.1. Demographic and Anthropometric Data between Mothers and Newborns with or without Preterm Delivery

After excluding twins, a total of 211 pregnant women (189 deliveries at term and 22 preterm) were enrolled in this study (Supplementary Figure S1). The mothers with sPTB and term birth were compared for height, body weight, and BMI before pregnancy. Complete blood counts with differential counts obtained in the second trimester were also compared. The age in weeks at delivery, birth body length, birth body weight, head circumference, chest circumference, and sex distribution were compared between preterm and term newborns (Table 1).

Compared to mothers with term delivery, mothers with sPTB showed significantly higher body weight and BMI before pregnancy. Mothers with preterm delivery also showed higher leukocyte counts than those with term birth (Table 1). There was no difference in delivery method and gender distribution between the preterm and term groups. The gestational age for the preterm and term infants was 34.45 ± 0.45 and 38.92 ± 0.08 weeks old, respectively. It is reasonable for preterm babies to have lower weight, height, head circumference, chest circumference, and abdominal circumference than babies born at term. With food frequency questionnaire analysis, mothers with preterm infants had only a higher cholesterol intake than mothers with term infants (Supplementary Table S1).

### 3.2. Gut Microbiota of Overweight Mothers before Pregnancy Is Dysregulated

To illustrate whether the gut microbiome is already undergoing dysbiosis before delivery in mothers who give preterm birth, we determined the proportions of 16S rDNA amplicons assigned to each phylum. Obesity is well known to be an important factor that affect gut microbiome. Since the mothers with preterm birth had a higher BMI before pregnancy than the mothers with term birth, the mothers were further divided into subgroups with normal BMI (18 < BMI < 24) and overweight group (BMI *≥* 24) according to their BW before pregnancy to control the effect of BMI on the gut microbiome. The demographic and anthropometric data of subgroups are shown in Table 2.

**Figure 1:**
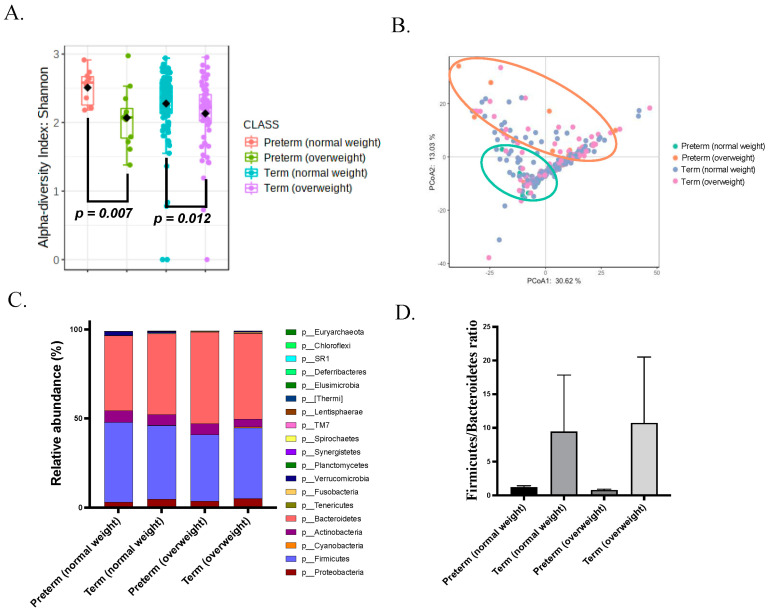
Gut microbiota alpha and beta diversity did not change in sPTB. (**A**) Shannon (richness and evenness) diversity index was significantly increased in overweight groups but did not change in sPTB. (**B**) Gut microbiota (Weighted UniFrac) composition did not show significant change in sPTB or overweight. (**C**) Top 10 phylum relative abundance analysis did not show significant change. (**D**) Firmicutes to Bacteroidetes ratio did not show significant change in sPTB and control. A *p* value less than 0.05 was considered as statistic significant.

The overweight group showed a lower α-diversity than the normal BMI group based on the Shannon index (both richness and evenness) in both preterm and term conditions (*p* = 0.007 and 0.012, respectively; Figure 1A). Since the *α*-diversity is the mean species diversity within a single sample, the gut microbiome of mothers with overweight before pregnancy has a relatively lower number and abundance of taxa than that of mothers with normal BMI. The *β*-diversity with the principal coordinate analysis (PCoA) of D_0.5 UniFrac was also analyzed, but the PERMANOVA test displayed no significant difference in the overall microbial composition between the normal BMI and overweight subgroups and between the sPTB and term groups (Figure 1B). An increased Firmicutes-to-Bacteroidetes ratio (F/B ratio), caused by an increase in the abundance of Firmicutes with or without a decrease in the abundance of Bacteroidetes, was previously found to be a biological signature of gut dysbiosis in metabolic disorders [33,34]. To determine whether preterm mothers have a similar gut microbiota composition, their F/B ratio was analyzed. Firmicutes was the most abundant phylum. The F/B ratio between mothers with preterm birth and mothers with term birth did not arrive statistically significance (Figure 1C,D).

### 3.3. Gut Dysbiosis in Mothers with sPTB before Delivery

**Figure 2:**
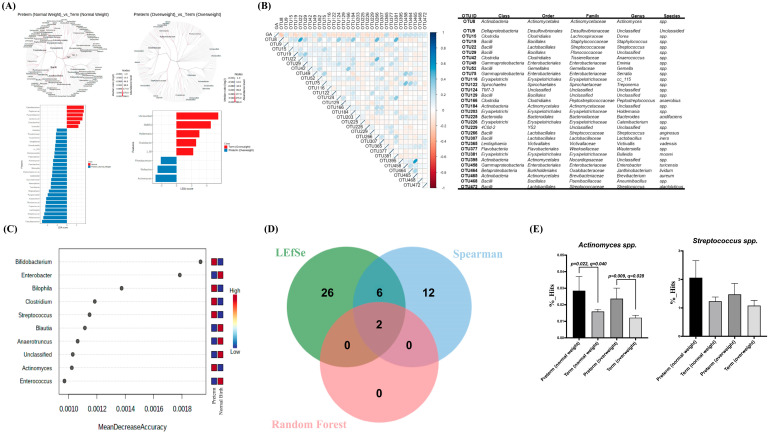
Core gut microbiota analysis in sPTB. (**A**) Core microbiota analysis by LEfSe (Linear discriminant analysis (LDA) Effect Size, LDA score > 2.0 and Wilcoxon *p* value cutoff = 0.05) in term vs. sPTB mothers with normal weight before pregnancy and (**B**) term vs. sPTB mothers with overweight before pregnancy. Critical gut microbiota which presents in term and sPTB form kingdom (center) to species (outer) level. The bar plots indicate critical gut microbiota in genus level in term and sPTB mothers with normal or overweight before pregnancy. (**C**) The correlation of gestational age (GA) to gut microbiota was analyzed by Spearman’s correlation. (**D**) Venn gram showing the union and the intersection of LEfSe, Spearman correlation, and random forest model. *Actinomyces* spp. and *Streptococcus* spp. were the only two species that belong the intersection. (**E**) Significant differences between *Actinomyces* spp. and *Streptococcus* spp. were validified by comparing the hits number with the *p* value less than 0.05 was considered as statistically significant. False discovery rate was used by the Benjamini–Hochberg methods and the q-value less than 0.05 was considered as statistically significant.

Linear discriminant analysis (LDA) effect size (LEfSe) analysis was used to distinguish the taxonomic abundance between mothers with preterm and term deliveries. In the cladogram obtained, branch extended from the inside to the outside represent the classification levels from kingdom to species level. In the LDA distribution histogram, the length of the histogram represents the size of the different species (i.e., LDA score). The bar-plot in left panel of Figure 2A illustrated the genera of gut microbiome between the mothers with sPTB of normal weight before pregnancy (in blue color) and the mothers with term delivery of normal weight before pregnancy (in red color). The top three genera in mothers with sPTB more than mothers with term delivery of normal weight before pregnancy was *Faecalibacterium*, *Akkermansia*, and *Streptococcus* spp. The genera difference between the mothers with sPTB of over-weight before pregnancy and the mothers with term delivery of over-weight before pregnancy is shown in the right panel of Figure 2A. The top three genera in mothers with sPTB more than mothers with term delivery of over-weight before pregnancy were *Actinomyces*, *Wolbachia*, and *Photobacterium* spp. (in blue color).

### 3.4. Association of Specific Microbial Biomarkers with sPTB Risk

Pregnancy is a continuous process. The correlation was further investigated for gestational age and the gut microbiome composition. A total of 485 bacteria were included in the analysis. A clustered heatmap of the Spearman correlation test is illustrated and validated (Figure 2B). The spectra that significantly correlated with gestational age were shown at the class, order, family, genus, and species levels. The abundance of *Actinomyces* spp. was all negatively correlated with gestational age at class, order, family, and genus level (r = −0.251, *p* < 0.001). More intriguingly, the random forest model also confirmed *Actinomyces* spp. is one of the critical gut microbes for sPTB (mean decrease accuracy = 0.002) and was the only taxa to have increased abundance in sPTB mothers compared to term mothers who were of standard weight and overweight before pregnancy (Figure 2C). *Actinomyces* spp. and *Streptococcus* spp. were the only two species belonging to the intersection of LEfSe, Spearman correlation, and random forest model (Figure 2D). Significant differences were then validified by comparing the hits number with *T*-test and we found Actinomyces was more abundant in mothers with sPTB than mothers with term delivery with both normal weight and over-weight before delivery (*p* = 0.022 (q = 0.040) and *p* = 0.009 (q = 0.028), respectively, Figure 2E).

**Figure 3:**
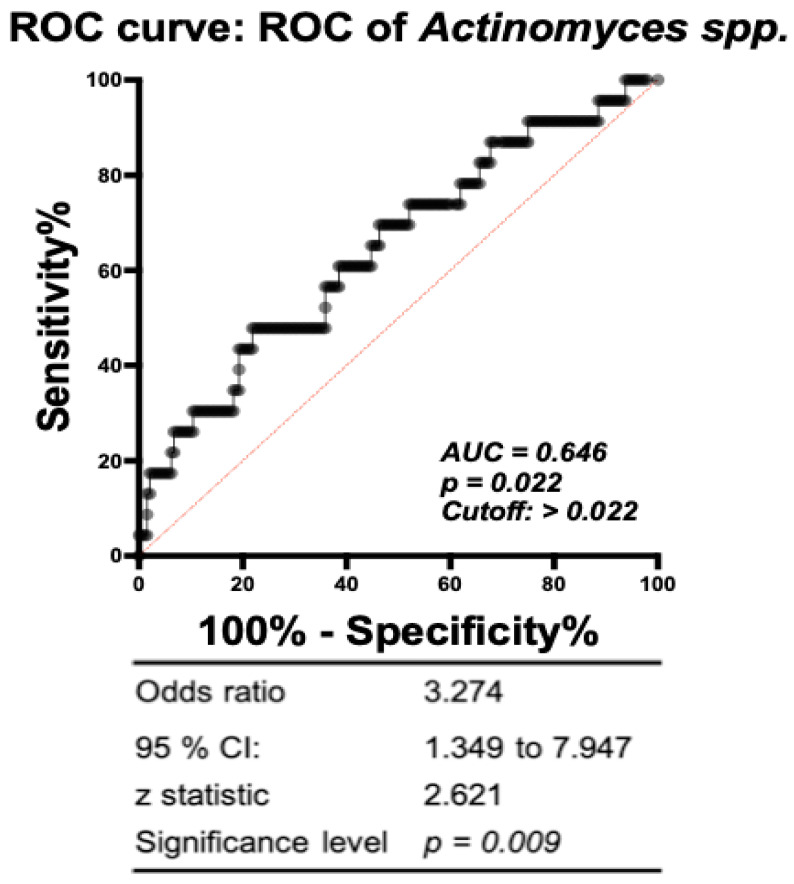
The discriminate predictive model indicated that *Actinomyces* spp. was a candidate to predict sPTB risk in all participants (AUC = 0.646, *p* = 0.022). ROC: receiver operating characteristic curve. AUC: area under the curve of ROC. *p* value less than 0.05 was considered statistically significant.

Since LEfSe analysis, association study, and random forest model all highlighted *Actinomyces* spp. highly related to the gut microbiome of mothers with sPTB, further work was conducted to determine whether the abundance of *Actinomyces* spp. in pregnant women could predict the occurrence of sPTB. A receiver operating characteristic (ROC) curve was used to analyze the diagnostic ability of *Actinomyces* spp. at the discrimination threshold. The area under the ROC curve for *Actinomyces* spp. was 0.646 (Figure 3). As for *Actinomyces* spp., when the cutoff value of the Hit% was set as 0.022, the hazard ratio for preterm delivery was 3.274 (95% CI: 1.349–7.947) for pregnant women.

### 3.5. Association of Potential Gut Dysbiosis-Associated Function Pathways with sPTB Risk

**Figure 4:**
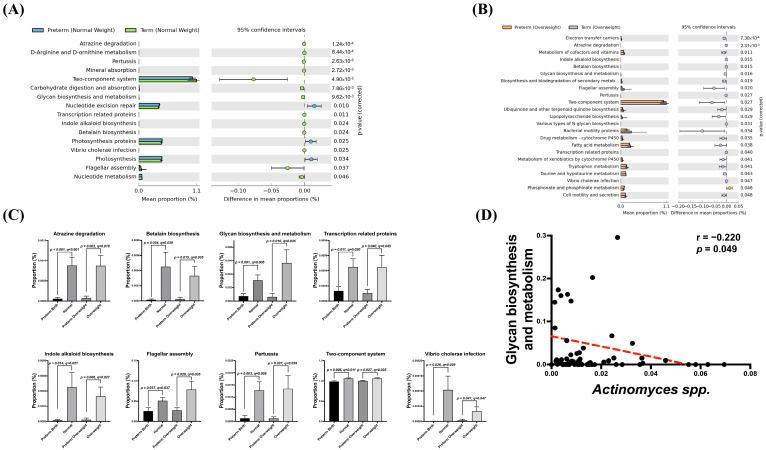
*Actinomyces* spp. was negatively correlated with glycan biosynthesis and metabolism. (**A**) Total of 16 functional pathways changed between normal weight with sPTB and controls by PICRUSt analysis. (**B**) Total of 23 functional pathways changed between overweight with sPTB and overweight control. (**C**) Total of 9 functional pathways were both changed in normal weight and overweight groups. (**D**) *Actionmyces* spp. was negatively correlated with glycan biosynthesis and metabolism (r = −0.220, *p* = 0.049). A *p* value less than 0.05 was considered statistically significant. False discovery rate was used by the Benjamini–Hochberg methods and the q-value less than 0.05 was considered statistically significant.

A gut microbiota-associated KEGG pathway enrichment analysis was conducted between mothers with preterm and term deliveries. There were 16 and 23 enriched pathways with significant differences between preterm and term mothers in the normal BMI and overweight subgroups, respectively (Figure 4A,B). A Venn diagram was used to determine the cross-functional pathways supposed to critically participate in sPTB occurrence. The expression of nine pathways, including those related to glycan biosynthesis and metabolism, betalain biosynthesis, atrazine metabolism, transcription-related proteins, flagellar assembly, pertussis, two-component system, Virbio cholerae infection, and indole alkaloid biosynthesis were increased in preterm mothers compared to those term mothers regardless of BMI (Figure 4C). To explore the association between the potential functional pathways of the predominant microbial markers and sPTB risk, the correlation of these nine consensus pathways and specific risk-associated gut microbes, such as *Actinomyces* spp., were analyzed using the Spearman correlation test. The glycan biosynthesis and metabolic pathway was the only pathway negatively correlated with the abundance of *Actinomyces* spp. (r = −0.220, *p* = 0.049) (Figure 4D). A multivariable correlation study did not reveal a significant association between nutrition intake and preterm risk. There was also no significant correlation between nutrition intake and gut microbiome.

## 4. Discussion

In this study, we compared the gut microbiome and dietary habits of mothers with preterm and term infants in the second trimester of pregnancy before delivery. We found that mothers with preterm infants had a higher BMI before pregnancy than those with infants born at term. Mothers with preterm infants also demonstrated a higher cholesterol intake than mothers with term infants. Gut microbiome analysis revealed gut dysbiosis in mothers with preterm delivery before parturition. With BMI correction, mothers with preterm delivery still showed a higher abundance of *Actinomyces* spp. than mothers with term delivery. In contrast, a study conducted in Norway showed that the gut microbiome of mothers who delivered premature infants showed a reduced diversity and lower abundance of *Bifidobacterium* and *Streptococcus* than that of term [28].

The causes of sPTB are still not clear, but are multifactorial. Four major mechanisms for preterm labor have been suggested: (1) maternal or fetal stress-induced premature activation of the fetal hypothalamic-pituitary-adrenal axis [35,36] (2) exaggerated inflammatory response or infection, (3) placental abruption, and (4) pathologic uterine distention. Epidemiologic studies have demonstrated an association between sPTB, the presence of systemic and genitourinary tract pathogens, and altered microbiomes and inflammatory responses [37,38,39,40,41,42]. Although delivery of live infants that occurs prior to the completion of 37 weeks of gestation is classified as preterm, pregnancy is a continuous process. Establishing a single cut-off point at 37 weeks of gestation, although classical, can be simplistic and debatable. Thus, the correlation between gestational age and maternal gut microbiome was analyzed with the Spearman correlation test in this study (Figure 2B). There is no strong evidence showing that antibiotic prophylaxis for pregnant women with asymptomatic bacterial vaginosis reduces their risk of sPTB [10,37,43]; it suggests that, in addition to the microbiome imbalance of the genital tract, there are other risk factors for preterm birth. Indeed, sPTB is also related to infections or inflammations outside the genital tract, such as periodontitis, urinary tract infections, and inflammatory bowel disease [44], indicating that spontaneous preterm birth may be closely related to gut inflammation. In general, the richness and diversity of the gut microbiome indicate a balanced metabolic status and resistance to pathogen attacks [45]. Our findings suggest that the low gut microbiome diversity in mothers with preterm delivery may be one of the underlying causes of the increased inflammation observed. Our results further revealed that the abundance of *Actinomyces* spp. was all negatively correlated with gestational age at class, order, family, and genus level. Recent studies have shown that even minor changes in the composition of a mother’s intestinal microbiota may be associated with an increased risk of sPTB. Shiozaki et al. showed that there are significantly low abundances of *Clostridium* subcluster XVIII, *Clostridium* cluster IV, *Clostridium* subcluster XIVa, and *Bacteroides*, and a significantly high abundance of *Lactobacillales* was observed in the intestinal microbiota of Japanese mothers in the sPTB group compared with those in the non-sPTB group using terminal restriction fragment length polymorphism (T-RFLP) analysis [29]. Japan S et al. noted that a decrease in α-diversity was strongly associated with the development of sPTB in the US, especially in the taxonomic class of *Betaproteobacteria* [46]. In the postpartum period, Dahl et al. found that mothers who delivered premature infants in Norway had a lower abundance of *Bifidobacterium*, *Streptococcus*, and genera in the order *Clostridiales* [28]. In another study, the gut microbiome of mothers who delivered premature babies in Finland had lower postpartum α- and β-diversity [47]. As regional and cultural factors have been shown to affect gut microbial diversity [48], further studies based on different ethnic backgrounds are warranted.

A previous study has shown that the efficiency of the gut microbiome in extracting energy from the diet is highly correlated with the expression of specific metabolic pathways [49]. An increase in the abundance of *Actinobacteria* was observed when comparing the third to the first trimester of pregnancy, showing the strongest signs of inflammation and energy loss [27]. *Actinomyces* are gram-positive bacteria that are anaerobic or facultative anaerobes. Most actinomycete species cluster together to form a structure similar to fungal colonies. *Actinomyces* are part of the normal flora in the vagina and oral cavity, but they can also infect the gastrointestinal tract, lungs, and uterus. *Actinomyces* can produce formate, acetate, succinate, lactate, and various antibiotics. The metabolic ability of *Actinomyces* may be due to the decomposition and recovery of organic compounds in the human gastrointestinal system [50]. In rare conditions, chorioamnionitis and preterm delivery can be complicated by *Actinomyces* infection [51].

In our study, several important metabolic pathways associated with gut dysbiosis in sPTB mothers were identified through functional assays. These pathways were associated with gut dysbiosis in mothers with preterm delivery, including atrazine degradation, betalain and glycan biosynthesis and metabolism, transcription-related proteins, and indole alkaloid biosynthesis. Some recent studies have suggested an association between preterm birth and atrazine, a commonly used herbicide [52,53,54]. *Actinomyces* are well-known environmental scrubbers that could release enzymes to clean up some pesticides, plastic products, and herbicides, including atrazine. The relationship between intestinal *Actinomyces*, atrazine, and preterm birth is intriguing. Another important signal is the tryptophan pathway (indole alkaloid biosynthesis). Karahoda et al. have shown that in the highly inflammatory type of preterm birth, levels of amniotic fluid IL-6 and maternal serum C-reactive protein are associated with changes in tryptophan catabolism [55]. Moreover, glycan biosynthesis and metabolism disorders have been found in carbohydrate imbalance and play important roles in weight control [56]. This study showed an imbalance in glycan biosynthesis and metabolism in the sPTB group. This result indicates that glycan might be an important nutrient during pregnancy.

Mothers who are overweight and obese during pregnancy have an increased risk of preterm delivery [57,58]. The risk of sPTB increase in obese women is currently attributed to their higher tissue inflammatory status [57]. Our results are in agreement with these previous findings. To control for confounding factors of obesity, we divided pregnant women into the normal weight and overweight subgroups according to their pre-pregnancy BMI. An increased abundance of *Actinomyces* spp. was found in both the normal-weight and overweight sPTB subgroups.

Our results highlight the clinical importance of maternal gut dysbiosis in preterm birth. The goal of this study was to assess whether maternal gut dysbiosis is already in existence before sPTB? Whether the occurrence of sPTB can be predicted from the maternal gut microbiome? In general, case-control study designs are not suitable for predicting the occurrence of disease. Case-control study designs are often used to investigate the association between disease and underlying risk factors, and often involve the use of already diseased individuals as research subjects. We therefore collected stool samples in the second trimester before sPTB and used a prospective study design to observe the temporal sequence between the maternal gut microbiome and the onset of sPTB. Because the analysis of gut microbiota is costly. There is a rare report in the literature about maternal gut microbiome predicting sPTB. Therefore, a calculation of the sample size necessary to obtain results on the expected objectives was not presented. We can only conduct a pilot study to collect data as far as possible with limited funding. However, we provided a valuable reference for further related studies.

The definition of overweight in Taiwan is ≥24 [59,60], that different from the classic WHO definition [61]. The difference in over-weight definition may due to differences in perceptions and assessments of the health risks of different BMI values in different countries. Taiwan is located in Asia, and its race, culture, etc. are different from Europe and the United States. Therefore, the impact of these factors may be taken into account when setting the BMI standard for defining overweight. This subject focus was on preterm birth, so we did not investigate the relationship between BMI as a continuous variable and the microbiota. Whether using a BMI ≥ 25 as a cutoff value would make a different result is a meaningful question and worthy of further investigation.

There are limitations to this study. First, the sample size of sPTB in this study is small, thus our work needs to be validated in larger studies. Besides the PICRUST is predictive of functional potential and more accurate functional profiling and metabolite analysis should be conducted. Given that preterm birth is a multifactorial entity, a single assessment of the maternal gut microbiota may be too risky to support a clear causal relationship. It could also explain the AUC not reaching 0.7 in Figure 3. Subsequent research on metabolites may be able to clarify the relationship between sPTB and the maternal gut microbiome.

## 5. Conclusions

Maternal gut microbiota of sPTB showed an increased abundance of *Actinomyces* spp., and dysregulated glycan metabolism may be associated with sPTB risk.

## Figures and Tables

**Table 1:** Demographic and anthropometric data of mothers and newborns with or without preterm delivery.

Parameters	Preterm	Term	*p*
Gestational Age	34.5 ± 0.5	38.9 ± 0.1	<0.001
Maternal Age	35.8 ± 0.7	35.3 ± 0.3	0.608
Maternal Height (cm)	160.4 ± 1.6	160.3 ± 0.4	0.925
Maternal Weight (Kg)	68.6 ± 3.2	59.7 ± 0.8	0.012
Maternal BMI	26.6 ± 1.2	23.2 ± 0.3	0.009
Parity	2.0 ± 0.2	1.9 ± 0.1	0.575
Glucose AC	136.7 ± 7.0	133.4 ± 2.3	0.655
Gestational DM	37%	35%	0.885
WBC (1000/μL)	12.4 ± 1.1	10.5 ± 0.3	0.040
RBC (million/μL)	4.2 ± 0.1	4.4 ± 0.2	0.745
Hemoglobin (g/dL)	11.6 ± 0.3	11.9 ± 0.1	0.475
MCV (fL)	86.3 ± 1.9	87.2 ± 0.6	0.622
Platelets (1000/μL)	239.5 ± 15.1	231.9 ± 4.1	0.562
Segment (%)	75.1 ± 2.1	76.5 ± 0.8	0.527
Lymphocyte (%)	17.7 ± 1.9	16.6 ± 0.6	0.542
Monocyte (%)	5.3 ± 0.4	5.3 ± 0.1	0.990
Eosinophil (%)	1.1 ± 0.2	0.8 ± 0.1	0.195
Basophil (%)	0.3 ± 0.1	0.3 ± 0.0	0.647
Delivery mode (CS/NSD)	10/12	57/132	0.280
Neonates data			
Neonatal Sex (M/F)	10/12	96/93	0.604
Neonatal Hight	46.4 ± 0.8	49.6 ± 0.2	0.001
Neonatal Weight	2394.8 ± 136.3	3152.83 ± 27.9	<0.001
Hand Circumference	31.2 ± 0.5	33.54 ± 0.1	<0.001
Chest Circumference	28.7 ± 0.6	32.51 ± 0.1	<0.001
Abdominal Circumference	26.5 ± 0.8	29.84 ± 0.1	<0.001

Abbreviation: body mass index, BMI; caesarean section, white blood cells, WBC; red blood cells, RBC; mean corpuscular volume, MCV; cesarean section, CS; normal spontaneous delivery, NSD; diabetes mellitus, DM; Head Circumference, HC; Chest Circumference, CC; Abdominal Circumference, AC; Glucose AC (Ante Cibum) means blood glucose before meal. The continuous variables were analyzed with Mann–Whitney U test.

**Table 2:** Anthropometric data of sub-grouped mothers and newborns with/without preterm delivery according to maternal BMI.

	Preterm		Term							
	BMI < 24	BMI ≥ 24	BMI < 24	BMI ≥ 24						
Group	1 (N = 10)	2 (N = 12)	3 (N = 123)	4 (N = 66)	1 vs. 2	1 vs. 3	1 vs. 4	2 vs. 3	2 vs. 4	3 vs. 4
Parameters	Mean ± SE	Mean ± SE	Mean ± SE	Mean ± SE	*p*-Value					
Maternal Height	160.9 ± 3.2	159.9 ± 1.4	160.7 ± 0.5	159.4 ± 0.7	0.768	0.947	0.658	0.667	0.769	0.154
Maternal Weight	57.1 ± 3.3	78.1 ± 3.1	54.3 ± 0.6	69.7 ± 1.2	<0.001	0.197	<0.001	<0.001	0.008	<0.001
Maternal Age	36.3 ± 1.0	35.4 ± 1.0	35.0 ± 0.4	36.0 ± 0.5	0.547	0.374	0.798	0.747	0.667	0.148
Maternal BMI	21.9 ± 0.6	30.5 ± 1.8	21.0 ± 0.2	27.4 ± 0.4	<0.001	0.135	<0.001	<0.001	0.003	<0.001
Parity	1.8 ± 0.3	2.2 ± 0.4	1.8 ± 0.1	2.0 ± 0.1	0.455	0.992	0.463	0.257	0.697	0.125
Glucose AC (mg/dl)	124.0 ± 7.2	150.8 ± 10.8	128.6 ± 2.4	142.3 ± 4.5	0.051	0.582	0.129	0.015	0.508	0.009
Gestational DM (N)	2	7	34	32	0.121	0.591	0.075	0.083	0.694	0.008
WBC (1000/μL)	12.6 ± 1.9	12.2 ± 1.4	10.8 ± 0.4	10.0 ± 0.4	0.883	0.218	0.046	0.247	0.138	0.181
RBC (million/μL)	4.1 ± 0.2	4.2 ± 0.2	4.2 ± 0.1	4.8 ± 0.6	0.572	0.748	0.649	0.576	0.683	0.130
Hemoglgbin (g/dL)	11.3 ± 0.6	11.8 ± 0.3	11.9 ± 0.2	11.9 ± 0.2	0.433	0.318	0.321	0.962	0.923	0.921
MCV (fL)	86.4 ± 3.8	86.3 ± 2.0	87.7 ± 0.7	86.4 ± 1.0	0.972	0.650	0.989	0.548	0.966	0.273
Platelets (1000/μL)	238.8 ± 30.8	240.1 ± 14.4	225.6 ± 5.1	243.7 ± 6.6	0.967	0.518	0.880	0.390	0.829	0.034
Segment (%)	78.5 ± 3.6	72.3 ± 2.5	78.4 ± 0.7	73.1 ± 1.8	0.157	0.975	0.285	0.013	0.854	0.001
Lymphocyte (%)	15.0 ± 3.0	19.6 ± 2.3	15.4 ± 0.6	18.8 ± 1.2	0.232	0.915	0.249	0.028	0.784	0.003
Monocyte (%)	5.1 ± 0.4	5.4 ± 0.6	5.2 ± 0.2	5.4 ± 0.2	0.644	0.820	0.555	0.727	0.941	0.547
Eosinophil (%)	0.9 ± 0.3	1.2 ± 0.2	0.7 ± 0.1	1.0 ± 0.1	0.327	0.590	0.720	0.034	0.438	0.042
Basophil (%)	0.2 ± 0.1	0.3 ± 0.1	0.3 ± 0.0	0.3 ± 0.0	0.330	0.665	0.539	0.249	0.398	0.614
Gestational Age	34.6 ± 0.8	34.3 ± 0.5	38.8 ± 0.1	39.1 ± 0.1	0.776	<0.001	<0.001	<0.001	<0.001	0.206
Neonatal Gender	0.6 ± 0.2	0.3 ± 0.1	0.5 ± 0.1	0.5 ± 0.1	0.230	0.738	0.397	0.179	0.446	0.239
Neonatal Height	45.6 ± 1.2	47.0 ± 1.1	49.6 ± 0.2	49.7 ± 0.3	0.387	<0.001	<0.001	0.001	0.001	0.775
Neonatal Weight	2249.0 ± 154.9	2516.3 ± 213.7	3098.7 ± 32.9	3253.8 ± 49.3	0.341	<0.001	<0.001	0.020	0.006	0.008
Neonatal HC	30.5 ± 0.8	31.8 ± 0.6	33.3 ± 0.1	34.0 ± 0.2	0.196	<0.001	<0.001	0.001	<0.001	0.002 *
Neonatal CC	28.1 ± 0.8	29.3 ± 0.9	32.3 ± 0.2	32.8 ± 0.2	0.346	<0.001	<0.001	0.005	0.002	0.056
Neonatal AC	25.7 ± 1.1	27.2 ± 1.0	29.7 ± 0.2	30.2 ± 0.2	0.347	0.007	0.003	0.038	0.018	0.096
APGAR (1 min)	8.4 ± 0.3	7.8 ± 0.4	8.9 ± 0.1	8.7 ± 0.1	0.336	0.165	0.367	0.032	0.074	0.180
APGAR (5 min)	9.6 ± 0.2	9.3 ± 0.3	10.0 ± 0.0	9.9 ± 0.1	0.481	0.131	0.227	0.048	0.077	0.223

The unit of gestational diabetes mellitus (DM) is case number. Glucose AC (Ante Cibum) means blood glucose before meal. * The continuous variables were analyzed with one-way ANOVA test.

## Data Availability

The data can be requested via contact the corresponding authors after publication.

## Supplementary Materials

The following supporting information can be downloaded at: https://www.mdpi.com/article/10.3390/microorganisms11051171/s1. Figure S1: Total eligible patients; Table S1: Dietary and Nutrition comparison between mothers with preterm and term.

## References

[1] Liu L., Oza S., Hogan D., Chu Y., Perin J., Zhu J., Lawn J.E., Cousens S., Mathers C., Black R.E. (2016). Global, regional, and national causes of under-5 mortality in 2000–15: An updated systematic analysis with implications for the Sustainable Development Goals. Lancet.

[2] Lawn J.E., Cousens S., Zupan J. (2005). 4 million neonatal deaths: When? Where? Why?. Lancet.

[3] Platt M.J. (2014). Outcomes in preterm infants. Public Health.

[4] Menon R. (2008). Spontaneous preterm birth, a clinical dilemma: Etiologic, pathophysiologic and genetic heterogeneities and racial disparity. Acta Obstet. Gynecol. Scand..

[5] Goldenberg R.L., Hauth J.C., Andrews W.W. (2000). Intrauterine Infection and Preterm Delivery. N. Engl. J. Med..

[6] Romero R., Espinoza J., Kusanovic J.P., Gotsch F., Hassan S., Erez O., Chaiworapongsa T., Mazor M. (2006). The preterm parturition syndrome. BJOG Int. J. Obstet. Gynaecol..

[7] Gravett M.G., Novy M.J., Rosenfeld R.G., Reddy A.P., Jacob T., Turner M., McCormack A., Lapidus J.A., Hitti J., Eschenbach D.A. (2004). Diagnosis of Intra-amniotic Infection by Proteomic Profiling and Identification of Novel Biomarkers. JAMA.

[8] Sweeney E.L., Kallapur S.G., Gisslen T., Lambers D.S., Chougnet C.A., Stephenson S.-A., Jobe A.H., Knox C.L. (2015). Placental Infection With *Ureaplasma* species Is Associated With Histologic Chorioamnionitis and Adverse Outcomes in Moderately Preterm and Late-Preterm Infants. J. Infect. Dis..

[9] Andrews W.W., Goldenberg R.L., Mercer B., Iams J., Meis P., Moawad A., Das A., VanDorsten J.P., Caritis S.N., Thurnau G. (2000). The Preterm Prediction Study: Association of second-trimester genitourinary chlamydia infection with subsequent spontaneous preterm birth. Am. J. Obstet. Gynecol..

[10] Kahwati L.C., Clark R., Berkman N., Urrutia R., Patel S.V., Zeng J., Viswanathan M. (2020). Screening for Bacterial Vaginosis in Pregnant Adolescents and Women to Prevent Preterm Delivery: Updated Evidence Report and Systematic Review for the US Preventive Services Task Force. JAMA.

[11] Pararas M.V., Skevaki C.L., Kafetzis D.A. (2006). Preterm birth due to maternal infection: Causative pathogens and modes of prevention. Eur. J. Clin. Microbiol. Infect. Dis..

[12] Andrews W.W., Klebanoff M.A., Thom E.A., Hauth J.C., Carey J.C., Meis P.J., Caritis S.N., Leveno K.J., Wapner R.J., Varner M.W. (2006). Midpregnancy genitourinary tract infection with Chlamydia trachomatis: Association with subsequent preterm delivery in women with bacterial vaginosis and Trichomonas vaginalis. Am. J. Obstet. Gynecol..

[13] Goldenberg R.L., Culhane J.F., Iams J.D., Romero R. (2008). Epidemiology and causes of preterm birth. Lancet.

[14] Bayar E., Bennett P.R., Chan D., Sykes L., MacIntyre D.A. (2020). The pregnancy microbiome and preterm birth. Semin. Immunopathol..

[15] Chu D.M., Seferovic M., Pace R.M., Aagaard K.M. (2018). The microbiome in preterm birth. Best Pract. Res. Clin. Obstet. Gynaecol..

[16] Mueller-Heubach E., Rubinstein D.N., Schwarz S.S. (1990). Histologic chorioamnionitis and preterm delivery in different patient populations. Obstet. Gynecol..

[17] Romero R., Avila C., Santhanam U., Sehgal P.B. (1990). Amniotic fluid interleukin 6 in preterm labor. Association with infection. J. Clin. Investig..

[18] O’hanlon D.E., Moench T.R., Cone R.A. (2013). Vaginal pH and Microbicidal Lactic Acid When Lactobacilli Dominate the Microbiota. PLoS ONE.

[19] Ravel J., Gajer P., Abdo Z., Schneider G.M., Koenig S.S.K., McCulle S.L., Karlebach S., Gorle R., Russell J., Tacket C.O. (2011). Vaginal microbiome of reproductive-age women. Proc. Natl. Acad. Sci. USA.

[20] Tortelli B.A., Lewis W.G., Allsworth J.E., Member-Meneh N., Foster L.R., Reno H.E., Peipert J.F., Fay J.C., Lewis A.L. (2020). Associations between the vaginal microbiome and Candida colonization in women of reproductive age. Am. J. Obstet. Gynecol..

[21] Fettweis J.M., Brooks J.P., Serrano M.G., Sheth N.U., Girerd P.H., Edwards D.J., Strauss J.F., Jefferson K.K., Buck G.A. (2014). The Vaginal Microbiome Consortium Differences in vaginal microbiome in African American women versus women of European ancestry. Microbiology.

[22] Fettweis J.M., Serrano M.G., Brooks J.P., Edwards D.J., Girerd P.H., Parikh H.I., Huang B., Arodz T.J., Edupuganti L., Glascock A.L. (2019). The vaginal microbiome and preterm birth. Nat. Med..

[23] Callahan B.J., DiGiulio D.B., Goltsman D.S.A., Sun C.L., Costello E.K., Jeganathan P., Biggio J.R., Wong R.J., Druzin M.L., Shaw G.M. (2017). Replication and refinement of a vaginal microbial signature of preterm birth in two racially distinct cohorts of US women. Proc. Natl. Acad. Sci..

[24] Kindinger L.M., Bennett P.R., Lee Y.S., Marchesi J.R., Smith A., Cacciatore S., Holmes E., Nicholson J.K., Teoh T.G., MacIntyre D.A. (2017). The interaction between vaginal microbiota, cervical length, and vaginal progesterone treatment for preterm birth risk. Microbiome.

[25] Donaldson G.P., Lee S.M., Mazmanian S.K. (2016). Gut biogeography of the bacterial microbiota. Nat. Rev. Microbiol..

[26] Dunlop A.L., Knight A.K., Satten G.A., Cutler A.J., Wright M.L., Mitchell R.M., Read T.D., Mulle J., Hertzberg V.S., Hill C.C. (2019). Stability of the vaginal, oral, and gut microbiota across pregnancy among African American women: The effect of socioeconomic status and antibiotic exposure. PeerJ.

[27] Koren O., Goodrich J.K., Cullender T.C., Spor A., Laitinen K., Bäckhed H.K., Gonzalez A., Werner J.J., Angenent L.T., Knight R. (2012). Host Remodeling of the Gut Microbiome and Metabolic Changes during Pregnancy. Cell.

[28] Dahl C., Stanislawski M., Iszatt N., Mandal S., Lozupone C., Clemente J.C., Knight R., Stigum H., Eggesbø M. (2017). Gut microbiome of mothers delivering prematurely shows reduced diversity and lower relative abundance of Bifidobacterium and Streptococcus. PLoS ONE.

[29] Shiozaki A., Yoneda S., Yoneda N., Yonezawa R., Matsubayashi T., Seo G., Saito S. (2014). Intestinal Microbiota is Different in Women with Preterm Birth: Results from Terminal Restriction Fragment Length Polymorphism Analysis. PLoS ONE.

[30] Shih C.-T., Yeh Y.-T., Lin C.-C., Yang L.-Y., Chiang C.-P. (2020). *Akkermansia muciniphila* is Negatively Correlated with Hemoglobin A1c in Refractory Diabetes. Microorganisms.

[31] Fu M.-L., Cheng L., Tu S.-H., Pan W.-H. (2007). Association between Unhealthful Eating Patterns and Unfavorable Overall School Performance in Children. J. Am. Diet. Assoc..

[32] Klindworth A., Pruesse E., Schweer T., Peplies J., Quast C., Horn M., Glöckner F.O. (2013). Evaluation of General 16S Ribosomal RNA Gene PCR Primers for Classical and Next-Generation Sequencing-Based Diversity Studies. Nucleic Acids Res..

[33] Magne F., Gotteland M., Gauthier L., Zazueta A., Pesoa S., Navarrete P., Balamurugan R. (2020). The Firmicutes/Bacteroidetes Ratio: A Relevant Marker of Gut Dysbiosis in Obese Patients?. Nutrients.

[34] Grigor’eva I.N. (2020). Gallstone Disease, Obesity and the Firmicutes/Bacteroidetes Ratio as a Possible Biomarker of Gut Dysbiosis. J. Pers. Med..

[35] Lima S.A.M., El Dib R.P., Rodrigues M.R.K., Ferraz G.A.R., Molina A.C., Neto C.A.P., De Lima M.A.F., Rudge M.V.C. (2018). Is the risk of low birth weight or preterm labor greater when maternal stress is experienced during pregnancy? A systematic review and meta-analysis of cohort studies. PLoS ONE.

[36] Kelly R., Holzman C., Senagore P., Wang J., Tian Y., Rahbar M.H., Chung H. (2009). Placental Vascular Pathology Findings and Pathways to Preterm Delivery. Am. J. Epidemiol..

[37] Donders G.G., Van Calsteren K., Bellen G., Reybrouck R., Van den Bosch T., Riphagen I., Van Lierde S. (2009). Predictive value for preterm birth of abnormal vaginal flora, bacterial vaginosis and aerobic vaginitis during the first trimester of pregnancy. BJOG Int. J. Obstet. Gynaecol..

[38] Sheiner E., Mazor-Drey E., Levy A. (2009). Asymptomatic bacteriuria during pregnancy. J. Matern. Neonatal Med..

[39] Smaill F.M., Vazquez J.C. (2019). Antibiotics for asymptomatic bacteriuria in pregnancy. Cochrane Database Syst. Rev..

[40] Elovitz M.A., Gajer P., Riis V., Brown A.G., Humphrys M.S., Holm J.B., Ravel J. (2019). Cervicovaginal microbiota and local immune response modulate the risk of spontaneous preterm delivery. Nat. Commun..

[41] Kumar M., Murugesan S., Singh P., Saadaoui M., Elhag D.A., Terranegra A., Kabeer B.S.A., Marr A.K., Kino T., Brummaier T. (2021). Vaginal Microbiota and Cytokine Levels Predict Preterm Delivery in Asian Women. Front. Cell. Infect. Microbiol..

[42] Ansari A., Bose S., You Y., Park S., Kim Y. (2021). Molecular Mechanism of Microbiota Metabolites in Preterm Birth: Pathological and Therapeutic Insights. Int. J. Mol. Sci..

[43] Thinkhamrop J., Hofmeyr G.J., Adetoro O., Lumbiganon P., Ota E. (2015). Antibiotic prophylaxis during the second and third trimester to reduce adverse pregnancy outcomes and morbidity. Cochrane Database Syst. Rev..

[44] Bröms G., Granath F., Linder M., Stephansson O., Elmberg M., Kieler H. (2014). Birth Outcomes in Women with Inflammatory Bowel Disease: Effects of disease activity and drug exposure. Inflamm. Bowel Dis..

[45] Jandhyala S.M., Talukdar R., Subramanyam C., Vuyyuru H., Sasikala M., Nageshwar Reddy D. (2015). Role of the normal gut microbiota. World J. Gastroenterol..

[46] Gershuni V., Li Y., Elovitz M., Li H., Wu G.D., Compher C.W. (2021). Maternal gut microbiota reflecting poor diet quality is associated with spontaneous preterm birth in a prospective cohort study. Am. J. Clin. Nutr..

[47] Hiltunen H., Collado M.C., Ollila H., Kolari T., Tölkkö S., Isolauri E., Salminen S., Rautava S. (2021). Spontaneous preterm delivery is reflected in both early neonatal and maternal gut microbiota. Pediatr. Res..

[48] Yatsunenko T., Rey F.E., Manary M.J., Trehan I., Dominguez-Bello M.G., Contreras M., Magris M., Hidalgo G., Baldassano R.N., Anokhin A.P. (2012). Human gut microbiome viewed across age and geography. Nature.

[49] Turnbaugh P.J., Ley R.E., Mahowald M.A., Magrini V., Mardis E.R., Gordon J.I. (2006). An Obesity-Associated Gut Microbiome with Increased Capacity for Energy Harvest. Nature.

[50] Hanning I., Diaz-Sanchez S. (2015). The functionality of the gastrointestinal microbiome in non-human animals. Microbiome.

[51] Estrada S.M., Magann E.F., Napolitano P.G. (2017). Actinomyces in Pregnancy: A Review of the Literature. Obstet. Gynecol. Surv..

[52] Villanueva C.M., Durand G., Coutté M.-B., Chevrier C., Cordier S. (2005). Atrazine in municipal drinking water and risk of low birth weight, preterm delivery, and small-for-gestational-age status. Occup. Environ. Med..

[53] Stayner L.T., Almberg K., Jones R., Graber J., Pedersen M., Turyk M. (2017). Atrazine and nitrate in drinking water and the risk of preterm delivery and low birth weight in four Midwestern states. Environ. Res..

[54] Rinsky J.L., Hopenhayn C., Golla V., Browning S., Bush H.M. (2012). Atrazine Exposure in Public Drinking Water and Preterm Birth. Public Health Rep..

[55] Karahoda R., Robles M., Marushka J., Stranik J., Abad C., Horackova H., Tebbens J.D., Vaillancourt C., Kacerovsky M., Staud F. (2021). Prenatal inflammation as a link between placental expression signature of tryptophan metabolism and preterm birth. Hum. Mol. Genet..

[56] Woting A., Blaut M. (2016). The Intestinal Microbiota in Metabolic Disease. Nutrients.

[57] Tersigni C., Neri C., D’Ippolito S., Garofalo S., Martino C., Lanzone A., Scambia G., Di Simone N. (2020). Impact of maternal obesity on the risk of preterm delivery: Insights into pathogenic mechanisms. J. Matern. Fetal Neonatal Med..

[58] Cnattingius S., Villamor E., Johansson S., Bonamy A.-K.E., Persson M., Wikström A.-K., Granath F. (2013). Maternal Obesity and Risk of Preterm Delivery. JAMA.

[59] Liang C., Lee P.-F., Yeh P.-C. (2022). Relationship between Regular Leisure-Time Physical Activity and Underweight and Overweight Status in Taiwanese Young Adults: A Cross-Sectional Study. Int. J. Environ. Res. Public Health.

[60] Huang K.-C. (2008). Obesity and its related diseases in Taiwan. Obes. Rev..

[61] World Health Organization (2000). Obesity: Preventing and managing the global epidemic. Report of a WHO consultation. World Health Organ. Tech. Rep. Ser..

