# Supplementary material for: A Higher Abundance of Actinomyces spp. in the Gut Is Associated with Spontaneous Preterm Birth
Source: Microorganisms. 2023 Apr 29;11(5):1171. doi: 10.3390/microorganisms11051171 (PMC10222247; doi:10.3390/microorganisms11051171)
Supplement: Supplementary file 1 [file microorganisms-11-01171-s001.zip › Supplementary table S1.pdf]

Supplementary Table S1. Dietary and Nutrition comparison between mothers with preterm and term

Item	Preterm	Term	P value
Calories (Kcal)	1690.70 ± 92.81	1627.28 ± 38.28	0.586
Protein (g)	64.96 ± 3.35	62.50 ± 1.45	0.576
fat (g)	54.27 ± 3.16	51.98 ± 1.86	0.680
Carbonhydrate (g)	238.78± 16.10	230.77 ± 5.41	0.633
Protein (%)	15.42 ± 0.57	15.39 ± 0.19	0.957
fat (%)	27.70 ± 1.71	27.0 ± 0.59	0.711
Carbonhydrate (%)	53.04 ± 2.80	54.84 ± 0.89	0.514
Vitamin A (μg)	682.26 ± 45.25	725.480 ± 23,13	0.401
Vitamin D (μg)	4.69 ± 0.31	4.99 ± 0.17	0.405
Vitamin E (mg)	7.13 ± 0.58	6.98 ± 0.27	0.851
Vitamin B1 (mg)	0.99 ± 0.05	0.96 ± 0.02	0.668
Vitamin B2 (mg)	1.37 ± 0.09	1.38 ± 0.04	0.930
Niacin (mg)	15.32 ± 1.00	16.07 ± 0.46	0.587
Vitamin B6 (mg)	1.20 ± 0.07	1.24 ± 0.03	0.687
Vitamin B12 (μg)	3.98 ± 0.32	4.45 ± 0.17	0.352
Vitamin C (mg)	124.03 ± 11.27	127.87 ± 4.04	0.757
Fiber (g)	13.24 ± 0.91	13.43 ± 0.40	0.876
Cholesterol (mg)	325.15 ± 28.67	271.71 ± 8.41	0.044
