# Supplementary figures and images for: A Higher Abundance of Actinomyces spp. in the Gut Is Associated with Spontaneous Preterm Birth
Source: Microorganisms. 2023 Apr 29;11(5):1171. doi: 10.3390/microorganisms11051171 (PMC10222247; doi:10.3390/microorganisms11051171)

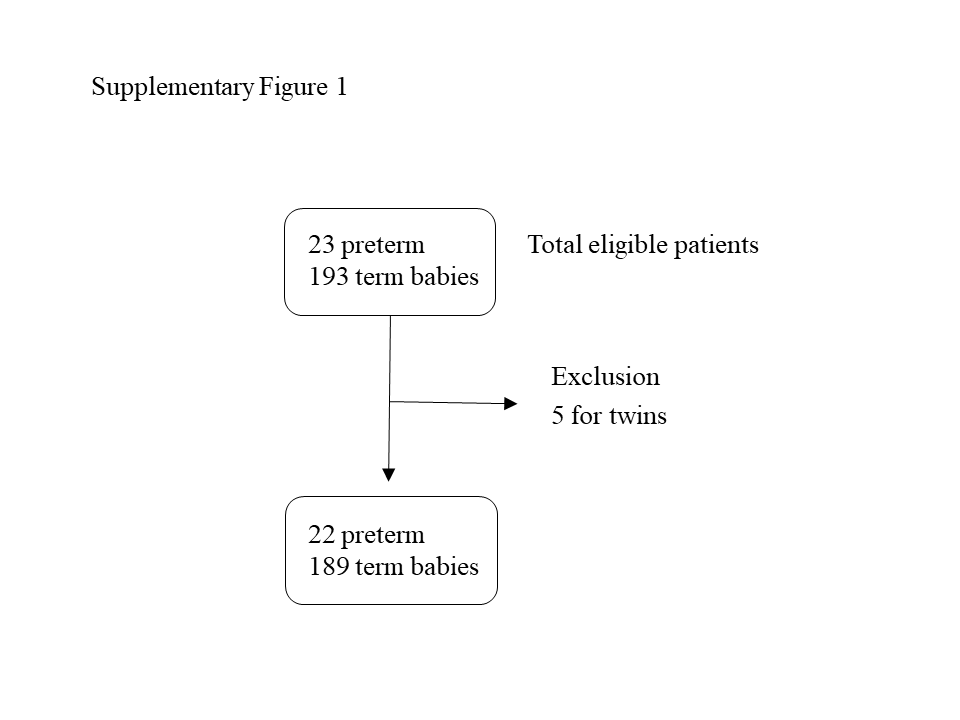

Supplement: Supplementary file 1 [file microorganisms-11-01171-s001.zip › Supplementary Figure S1.tif]
